# P-1643. Simply Scripting Success: Impact of Epic Preference List Changes on Antibiotic Prescribing in the Outpatient Setting

**DOI:** 10.1093/ofid/ofae631.1809

**Published:** 2025-01-29

**Authors:** Mary Smith, Amy Chang, David R Ha, Marisa Holubar, John Shepard, Emily Mui, William Alegria

**Affiliations:** Stanford Healthcare and Palo Alto Veterans Hospital , Santa Clara, California; Stanford University, Stanford, CA; Stanford Health Care, Stanford, CA; Stanford University School of Medicine, Stanford, CA; Stanford University, Stanford, CA; Stanford Health Care, Stanford, CA; Stanford Health Care, Stanford University School of Medicine, Stanford, California

## Abstract

**Background:**

Preference lists in electronic health records are commonly used to streamline ordering processes, including antibiotic orders. In July 2023, we revised our institution's primary care preference list to align with institutional guidelines for the management of skin and soft tissue infections (SSTI) and pneumonia (Figure 1). Here we evaluate the impact of this intervention on antibiotic prescribing practices.

**Figure:**
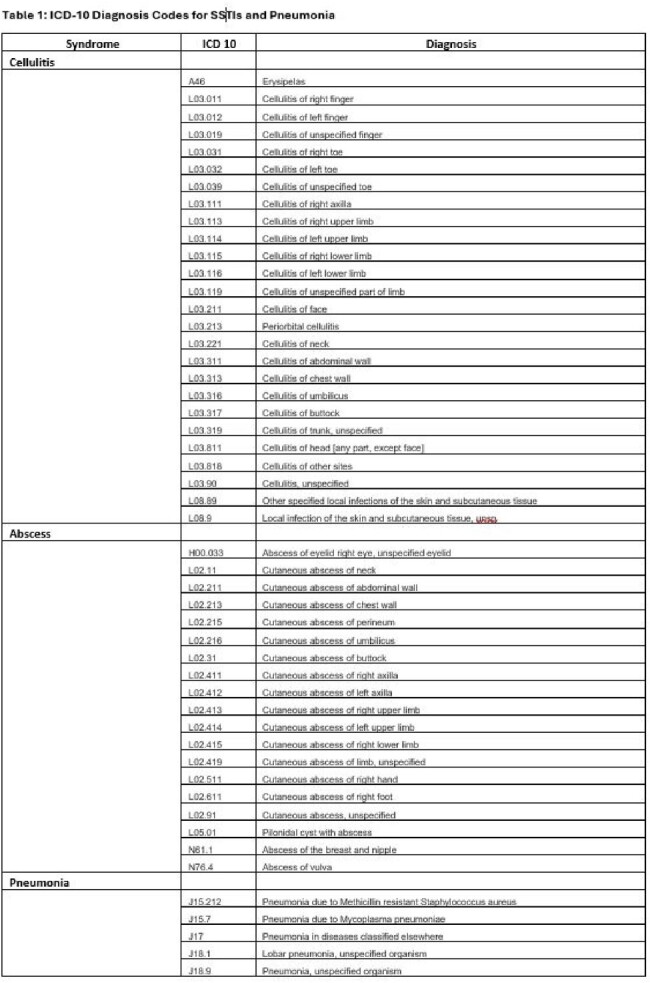

**Methods:**

We included all adult telemedicine and office visits associated with antibiotic orders from 2/2023 to 2/2024 at 8 academic primary clinics. We used International Classification of Diseases, 10^th^ revision (ICD10) data to identify encounters for SSTIs and pneumonia (Table 1 ). We defined “pre-intervention” as 2/1 – 7/31/2023 and “post-intervention” as 8/1/2023– 2/28/2024. We extracted encounter date, location, order source, antibiotic selection, and duration. We defined guideline adherence as 1^st^ line antibiotic selection and 5-day duration (Figure 1). There were no education or dissemination efforts to providers. This was deemed a non-human subjects research quality improvement project.

**Figure:**
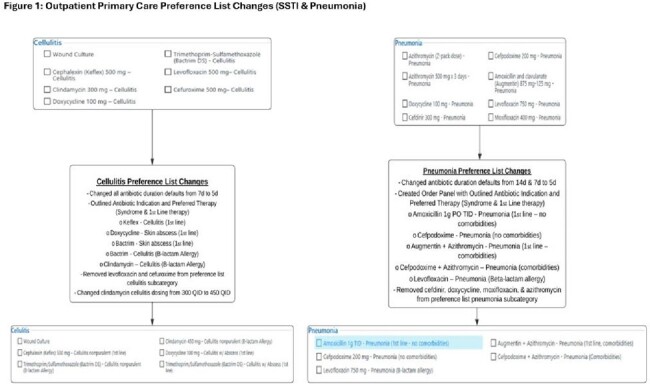

**Results:**

We included 349 total (165 pre, 184 post) encounters for SSTI (263) and pneumonia (86). 102 (29%) were telemedicine (50 pre, 52 post). Of the 349 encounters, 73 % of antibiotic orders were from the preference list (136 pre; 117 post). Guideline concordant antibiotic selection improved in the post-intervention group for pneumonia (8% pre, 44% post) and SSTIs (47% pre, 58% post), but only for orders sourced from the preference list (Table 2, Graphs 1-2). There was also improvement in guideline concordant 5-day durations with preference list orders post-intervention for pneumonia (55% pre, 78% post) and SSTI (20% pre, 50% post) (Table 2, Graphs 1-2). Antibiotic orders not using the preference list for these syndromes showed no improvement during this time.

**Figure:**
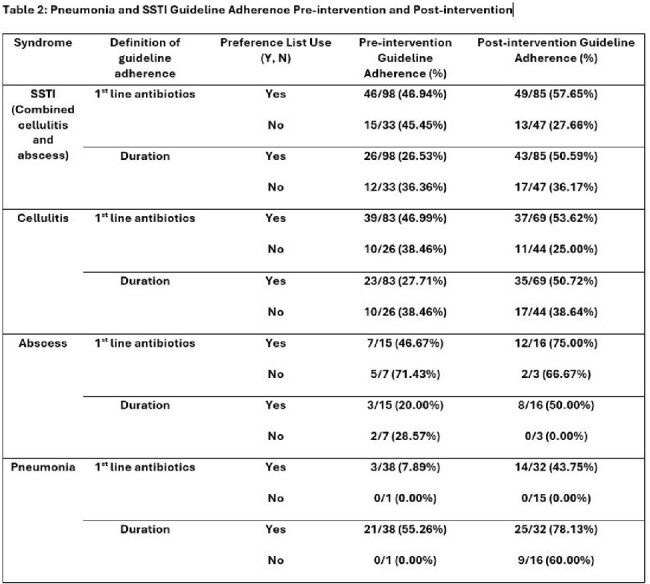

**Conclusion:**

We found that a simple revision of the primary care order preference list led to improved guideline concordant antibiotic prescribing for SSTI and pneumonia. For common syndromes in which antibiotic selection is typically made empirically in clinic, like SSTIs and pneumonia, pre-selected durations and highlighted first-line selections for antibiotic orders can improve adherence to guideline recommendations.

**Figure:**
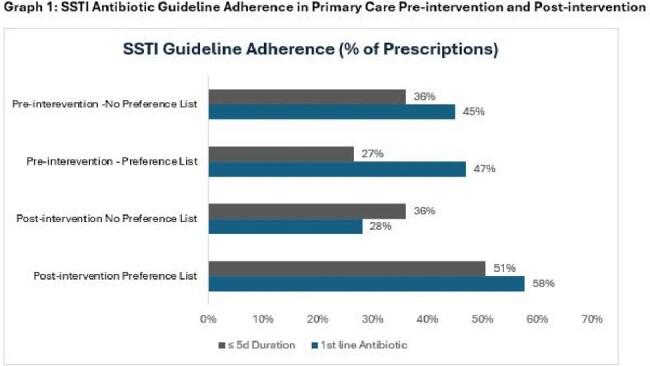

**Disclosures:**

**All Authors**: No reported disclosures

